# Accuracy of screw placement during vertebral body tethering using fluoroscopic guidance and anatomic landmarks

**DOI:** 10.1007/s43390-024-00970-4

**Published:** 2024-09-18

**Authors:** Kevin M. Neal, Kylie Krombholz, Mona Doshi

**Affiliations:** 1 Orthopaedics Department, Nemours Children’s Health, Jacksonville, FL USA; 2 Florida State University College of Medicine, Tallahassee, FL USA; 3 Orthopaedics Department, Nemours Children’s Health, Orlando, FL USA

**Keywords:** Vertebral body tethering, Screw accuracy, Image navigation, Spinal canal penetration

## Abstract

**Purpose:**

To determine the accuracy of screw placement using fluoroscopy and anatomic landmarks during vertebral body tethering (VBT) surgery.

**Methods:**

Ten patients with 73 VBT screws were converted to posterior spinal fusion (PSF) after continued curve progression. The positions of each VBT screw were analyzed using intraoperative computed tomography (CT) scans performed for image guidance during VBT. Differences for screws placed using an open versus thoracoscopic approach were noted for the screw position in each vertebra, distance from the spinal canal, unicortical versus bicortical placement, the distance of screw tips from the thoracic aorta, and impingement of screws on adjacent rib heads.

**Results:**

Seventy three (73) screws in ten (10) patients were available for analysis. Only 21% of screws were placed traversing the middle one-third of the vertebral body, without spinal canal penetration, with the distal tip placed unicortically or bicortically as planned, and without touching the thoracic aorta. The rates of non-ideal screw placement were not significantly different for screws placed via thoracoscopic versus open approaches. Five (5) screws (6.8%) penetrated the spinal canal 1–2 mm, but without known clinical sequelae.

**Conclusion:**

The majority of VBT screws available for analysis were placed in non-ideal positions, suggesting that accurate screw placement using intraoperative fluoroscopy and anatomic landmarks can be challenging, but without adverse clinical consequences.

## Introduction

Vertebral body tethering (VBT) is a surgical technique for fusionless correction of adolescent idiopathic scoliosis (AIS) that has gained popularity over the past decade [1–3]. During a VBT procedure, a tensioned rope is anchored to anterior vertebral bodies to correct spinal curvature and modulate growth [4]. While patients are in the lateral decubitus position, screws are typically placed in the thoracic spine using a thoracoscopic approach or in the lumbar spine using an open approach [4]. Most surgeons use adjunctive intraoperative fluoroscopy and visual landmarks to orient the trajectory of the screws correctly in the vertebral bodies.

The ideal trajectory for VBT screws is transverse across the middle one-third of each vertebral body, allowing sufficient bone for adequate screw purchase while avoiding the spinal canal. Placing screws in an ideal trajectory may be difficult due to the three-dimensional nature of scoliosis, including rotational deformity, coronal plane bending, and vertebral body dysplasia, especially at the apices of curves [5]. In addition to the variability of vertebral body morphology, other factors that may lead to non-ideal screw trajectories are difficulty in interpreting fluoroscopy, especially in the upper thoracic spine where the ribs, scapulae, or proximal humerus make fluoroscopic interpretation more difficult, or inadvertent movement of the patient’s thorax during the procedure, leading to distortion of planned visual landmarks. This study assesses the accuracy of screw placement using visual landmarks and adjunctive fluoroscopy during VBT procedures by evaluating actual screw positions seen on subsequent computed tomography (CT) scans of the same patients.

## Methods

This study is a retrospective review of patients who underwent a VBT procedure and had a subsequent CT scan of the included vertebral segments during conversion to posterior spinal fusion (PSF). Patients were identified from an existing, Institutional Review Board (IRB)-approved, prospective scoliosis database. A separate IRB-approval was obtained to review and analyze this specific dataset.

**Fig. 1:**
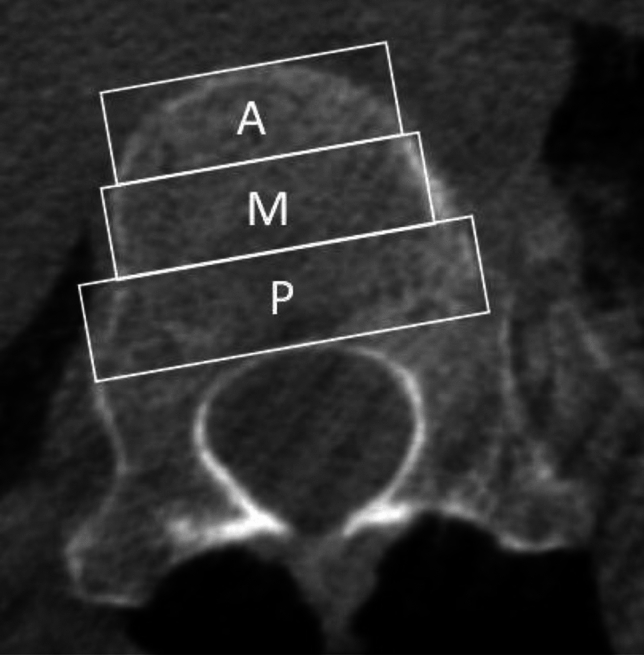
Division of vertebral bodies into anterior (A), middle (M), and posterior (P) thirds on axial CT images to document screw-starting and -ending points

Each screw trajectory was analyzed using multiple criteria. Each vertebral body in which a screw was placed was recorded. The approach used for screw placement (open versus thoracoscopic) and the laterality of each starting point (right versus left) were noted. The vertebral bodies were divided into thirds (anterior, middle, and posterior) and the starting and ending points of each screw were recorded, with the ideal placement defined as starting and ending points in the middle third (Fig. 1).

**Fig. 2:**
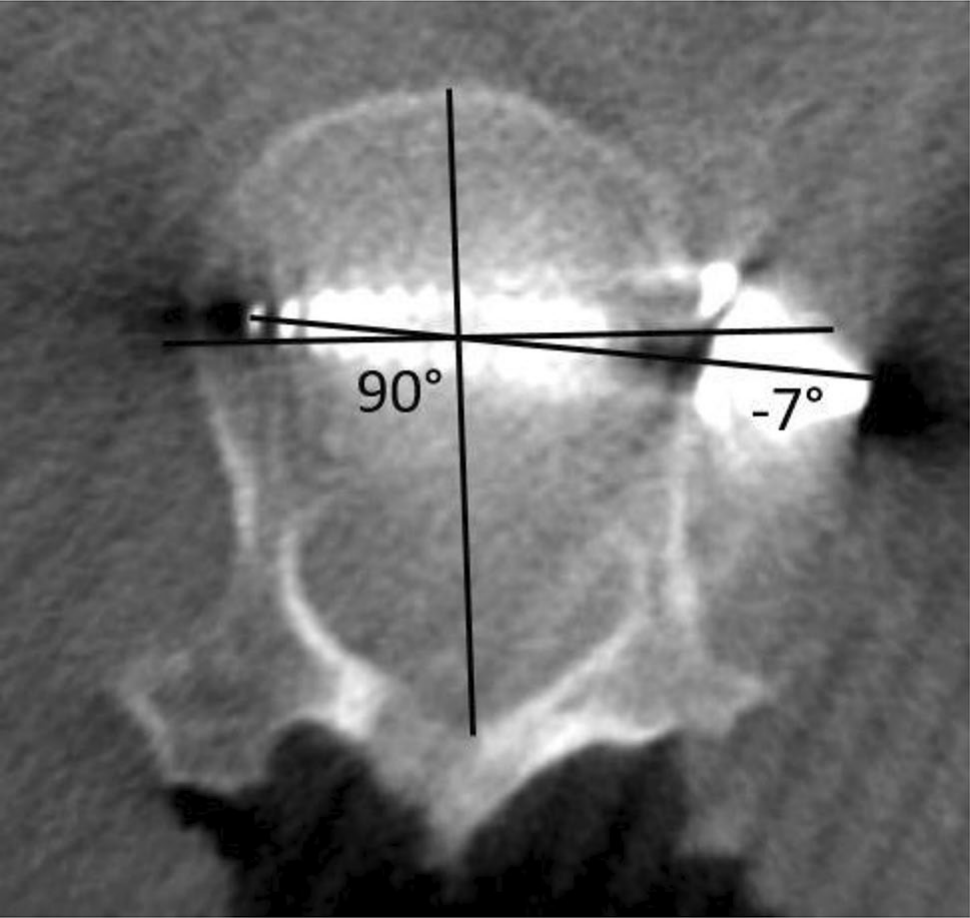
Axial CT image showing a line perpendicular to the long axis of the vertebral body, and a line through the long axis of the visualized screw with a screw angle of − 7° posterior to anterior

**Fig. 3:**
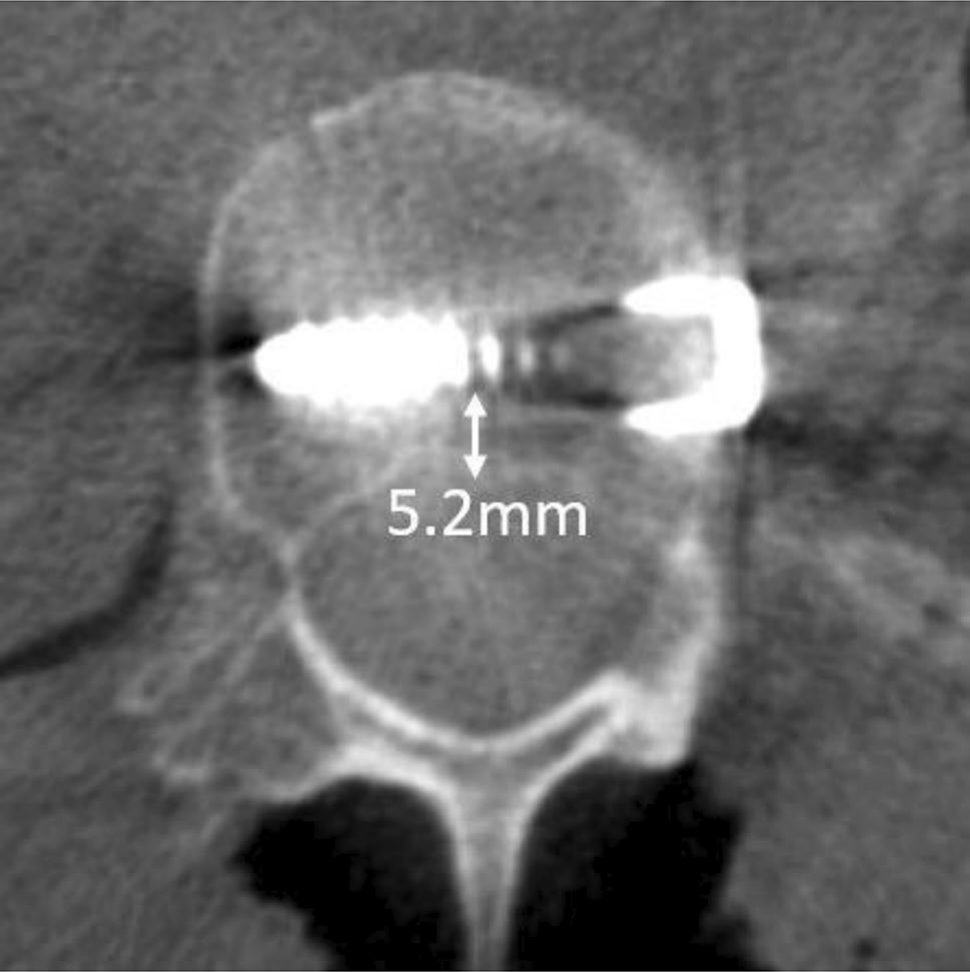
Axial CT image showing measurement of the closest distance of a screw to the spinal canal

**Fig. 4:**
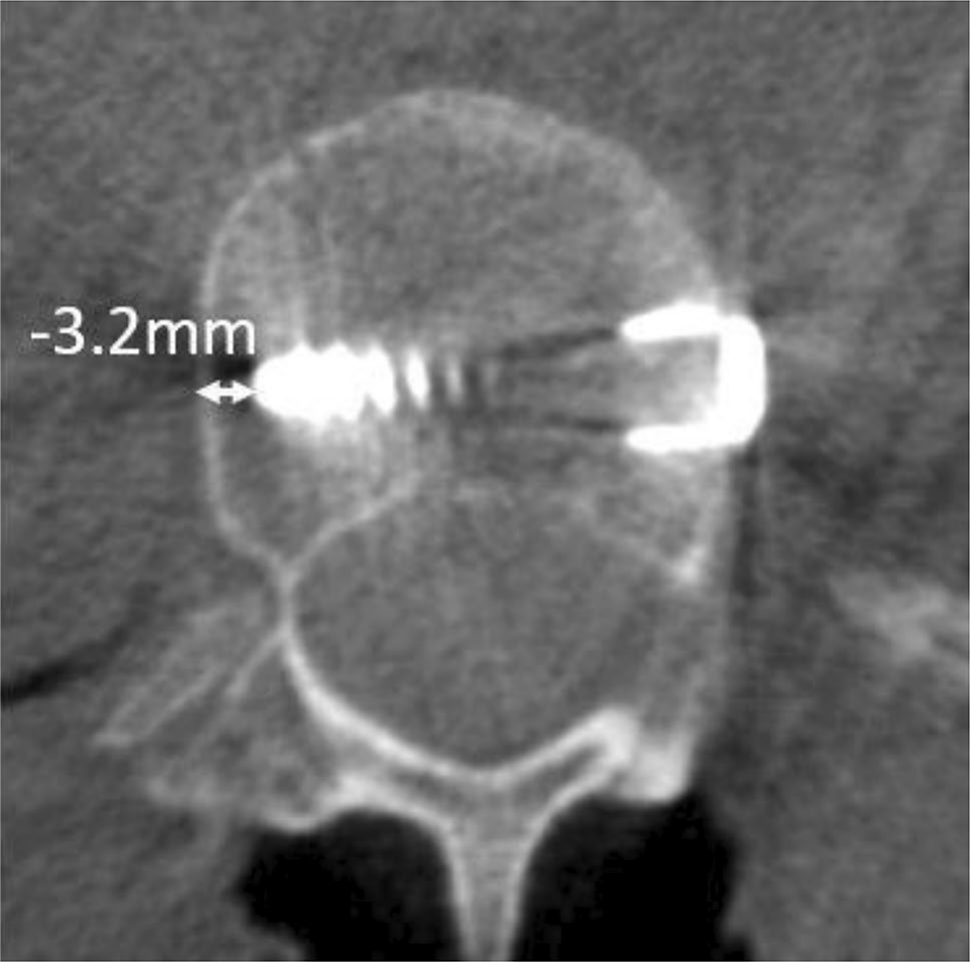
Axial CT image showing measurement of the distance from the tip of a screw to the far cortex of the vertebral body

**Fig. 5:**
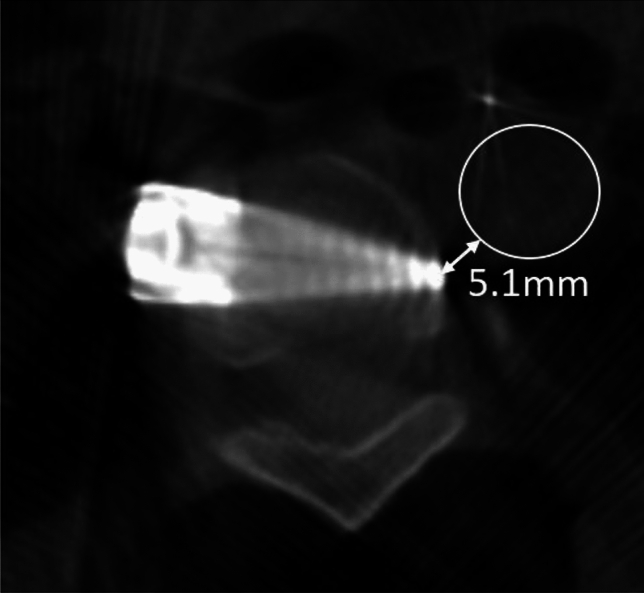
Axial CT image showing the measurement of the closest distance from the tip of a screw to the thoracic aorta (circle)

**Fig. 6:**
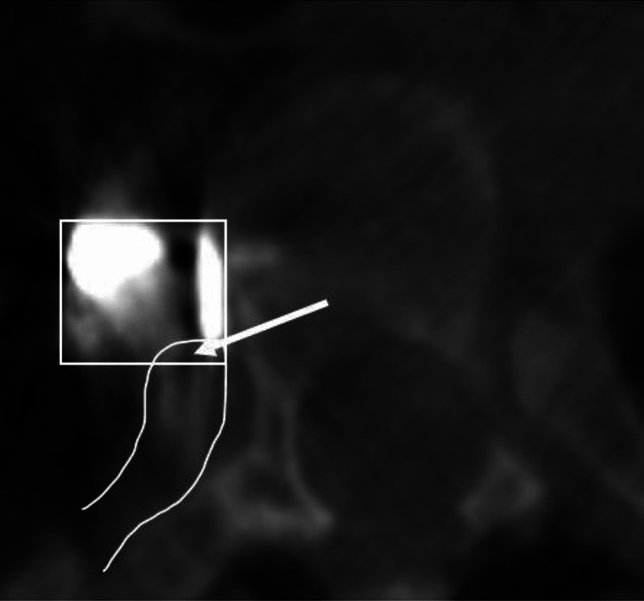
Axial CT image showing documentation of the head of a VBT screw (square) at its insertion point impinging on an outlined thoracic rib head

To measure the angle of screw placement, the angle between the actual screw trajectory and a line perpendicular to the long axis of the vertebral body was measured, with perfect placement defined as 0°. The angles of screws with anterior to posterior trajectories were recorded as positive and the angles of screws with posterior to anterior trajectories were recorded as negative (Fig. 2). The closest distance of any portion of each screw from the spinal canal was measured and any spinal canal breaches were recorded. The distance to the spinal canal was recorded as positive for screws that did not penetrate the canal, and negative for screws that entered the canal (Fig. 3). The screws were categorized as unicortical or bicortical. A screw was considered unicortical if the distal portion remained completely within the vertebral body, and bicortical if the screw tip penetrated the far cortex of the vertebral body. If the screw was unicortical, the distance of the screw tip to the far cortex was recorded as a negative number. If the screw was bicortical, the distance from the far cortex to the screw tip was recorded as a positive number (Fig. 4). For bicortical, thoracic screws, the distance from the screw tip to the aorta was recorded (Fig. 5). Any screw-head to rib-head impingement was noted for thoracic screws (Fig. 6).

Statistical analysis was perform using Chi-Square tests for nominal data and Student’s *t* test for independent means for interval numerical data. Calculations were performed at www.socialstatistics.com.

## Results

Fifty one (51) patients with minimum 2-year follow-up have undergone VBT surgery at our institution. Ten (10) patients (19.6%) have had subsequent curve progression requiring conversion to PSF using CT-image guidance. There were 73 screws placed in those ten (10) patients available for analysis. All patients were female with an average age of 11.1 years at the time of VBT and 14.1 years at the time of PSF. Twenty two (22) screws were placed in thoracic or lumbar vertebrae through an open approach for VBT, all with left-sided starting points. Fifty one (51) screws were placed in thoracic vertebrae with right-sided starting points using a thoracoscopic approach for VBT *(*Table 1*)*.

**Table 1:** Characteristics of 73 screws visualized on subsequent CT scans s/p VBT

73 screws	Thoracoscopic (*N* = 51)	Open (*N* = 22)	*p*
Thoracic vertebrae	51 (100%)	9 (40.9%)	
Middle star and end points	14 (27.5%)	13 (59%)	0.08
Mean screw angle	− 9°	− 2°	0.07
Spinal canal penetration	4 (7.8%)	1 (4.5%)	0.61
Mean distance to spinal cord	6 mm	7 mm	0.27

Overall, 27 of 73 screws (37.0%) had ideal middle starting and ending points. Screws were more likely to have ideal starting and end points when placed through an open approach (13 of 22) versus a thoracoscopic approach (14 of 51), however this difference was not statistically significant (*p* = 0.08). The mean screw angle was −2° (range −24° to 62°), posterior to anterior, for screws placed through an open approach versus −9° (range −33° to 24°) for screws placed using a thoracoscopic approach. This difference also did not reach statistical significance (*p* = 0.07).

Fifty nine (59) screws in eight patients were planned as unicortical screws and 14 screws in two patients were planned as bicortical screws. Overall, 41 screws were placed unicortically, and 32 screws were placed bicortically. Three (3) of 14 (21%) planned bicortical screws were placed unicortically. Twenty one (21) of 59 (35.6%) planned unicortical screws were placed bicortically. There was no statistical difference in the rate of unplanned placement between those with planned unicortical versus bicortical screws (*p* = 0.31). Of the screws placed bicortically, the average distance of the screw tip to the aorta was 12 mm, with one screw touching but not indenting the aorta. Five (6.8%) screws, 1 of 22 (4.5%) in the open-approach group and 4 of 51 (7.8%) in the thoracoscopic group, penetrated 1–2 mm into the spinal canal, (*p* = 0.61). The mean distance of any portion of each screw to the spinal canal was 7 mm (range −1 mm to 17 mm) in the open-approach group and 6 mm (range -2 mm to 15 mm) in the thoracoscopic group (*p* = 0.27). No thoracic screws in the open-approach group impinged on the rib heads. Only 9 of the 22 (41%) screws placed through an open approach were in thoracic vertebrae. Four (4) of 51 (7.8%) screws placed using a thoracoscopic approach impinged on rib heads. There were no cases of intraoperative neuromonitoring changes or postoperative neurologic deficits.

Fifteen (15) of 73 (21%) screws (7 of 22 (31.8%) in the open-approach group and 8 of 51 (15.7%) in the thoracoscopic group) had 1) starting points and ending points in the middle third of the vertebral bodies, 2) were placed unicortically or bicortically as planned, 3) did not touch the thoracic aorta, 4) had no spinal canal penetration, and 5) no rib-head impingement, (*p* = 0.12).

## Discussion

Our study provides insight into the difficulty of achieving ideal screw placement in vertebral body tethering procedures. Of the 73 screws analyzed in this study only 21% of those screws were placed in an ideal position: defined as (1) the screw starting just anterior to the convex rib head and traversing the middle one-third of the vertebral body perpendicular to the vertebral body bisector, (2) without spinal canal penetration, (3) with the distal tip placed unicortically or bicortically as planned, and (4) without touching the thoracic aorta. To our knowledge, this is the first study to document intraoperative accuracy of screw trajectories in VBT procedures.

Most studies to date have focused on short to medium term outcomes of VBT surgery. Hoerschemeyer et al. studied the postoperative radiographic and clinical outcomes of 29 individuals, 10–16 years of age who underwent a VBT [1]. They found that six (6) out of the 29 individuals needed a surgical revision, but of those only two underwent a PSF [1]. They did not note any instances of screw misplacement and their patients did not have CT scans at the time of PSF. Raitio et al. performed a systemic review of 843 VBT patients and found that 10% of the cases required reoperations and only 4.7% of the patients converted to PSF [6]. Similarly, they did not document any screw misplacement or findings of intraoperative CT scans at the time of PSF.

Newton et al. noted that individuals who underwent VBT continued to have significant spinal curvatures averaging 33° ± 18° compared with curvatures of 16° ± 6° in the PSF group [2]. They found that postoperative revisions were more likely in the individuals who underwent VBT as compared to those who underwent PSF. Nine (9) of their 23 VBT patients required revisions versus none in the 26 PSF patients. No screw misplacements were documented in their VBT or PSF groups, and CT data were not available for the three (3) revision patients who underwent PSF.

Screw misplacement has been previously reported during VBT procedures, however to our knowledge, this is the first attempt to directly analyze the intraoperative accuracy of screw trajectories. In their study of perioperative morbidity on 120 patients with 2-year follow-up Abdullah, et al. reported three cases of likely aberrant screw trajectories (2.5%) [7]. There were two cases of cerebrospinal fluid (CSF) leakage, one managed with a blood patch within 90 days of surgery, and one other that persisted despite a blood patch, eventually requiring a repeat surgery with a screw revision. They also noted one patient with leg paresthesias 2 years after surgery who was being managed conservatively. Though not explicitly stated, these complications were likely due to inappropriate screw trajectories with breaches of the spinal canal. Rushton, et al. reported two CSF leaks among 116 VBT surgeries with 2-year follow-up (1.7%) [8]. Both were recognized 1–2 weeks after the index procedures. The first was noted to be due to a screw breaching the spinal canal, while the cause for the second remained undetermined. Meyer, et al. reported complications on 184 VBT patients within 90 days of surgery [9]. The authors noted one patient who required a revision surgery due to a misplaced lumbar screw (0.5%). Our results showed a slightly higher percentage of spinal canal penetration (6.8%) compared to these prior studies, but did not find any cases of CSF leakage, paresthesias or revision surgery related to screw misplacement.

Because VBT is a relatively new technology, techniques for screw placement likely vary among participating centers. The most cited standard technique remains that described by Parent, et al. in 2020, using direct visualization and adjunctive fluoroscopic guidance [4]. In 2021, Mathew et al. described the technique of using CT-guided intraoperative image navigation to place VBT screws [10]. They performed VBT surgery on 67 patients using CT image guidance and compared operative outcomes for the first and last 20 patients. As their technique used image guidance for the placement of VBT screws, no subsequent CT scans were performed to document screw placement. No studies to date have described the use of intraoperative CT image guidance to check screw positioning intraoperatively. However, the success of their technique suggests it may be possible to develop protocols both for navigated screw placement, and to document appropriate screw positions.

Our study has several limitations. In addition to its retrospective design, we were only able to obtain data on patients who had curve progression despite VBT surgery and required a subsequent PSF using CT-guided image navigation. Forty one (41) patients who did not require subsequent PSF did not have CT scans performed, and no routine postoperative CT scans were performed after the initial VBT surgeries to document actual screw trajectories in those patients. Therefore, we are unable to determine if patients who require subsequent PSF after VBT might have a higher incidence of misplaced screws, compared to those who do not require PSF. Though our study cannot deduce whether the position of VBT screws may affect the rate of revision surgery, we suspect that this is unlikely since most screws in our study, even if not in ideal trajectories, still maintained adequate bony purchase. Because our study did not include clinical follow-up data or a control group for patients with nonideal screw placements, we are unable to document any differences in patient outcomes based on screw placement. Additional limitations include that screws were placed using both thoracoscopic and open approaches. Though there were several areas of variability between these groups that trended toward significance, it is likely that our study was underpowered to detect actual differences between those techniques.

We are aware of one patient in our database that had a CSF leak from a misplaced VBT screw, but who was not included because no subsequent CT scan was performed. In this case, screw penetration of the spinal canal was detected by intraoperative neuromonitoring. The patient lost both transcranial motor evoked potential (TcMEPs) and somatosensory evoked potentials (SSEPs) following screw placement. As part of the acute management, the implants were removed prior to any opportunity to perform an intraoperative CT scan to document the aberrant screw position. TcMEPs and SSEPs returned to baseline following implant removal. The patient awoke without any motor deficit, but with some contralateral decreases in temperature sensation (Brown-Séquard). Postoperative MRI scans were performed, showing a CSF leak and a pleural effusion, but the quality of the bony imaging was not sufficient to measure the trajectories of the former screw positions. The patient had seven screws placed before removal. Had these screws been included in our series, we would have recorded six total screws with spinal canal penetration, increasing the percentage of known spinal canal penetration from 6.8% to 7.5%.

## Conclusion/Summary

In our retrospective review of patients who went under VBT but then needed a PSF, we found that only 21% of screws in this cohort were placed in ideal positions, suggesting that screw placement using anatomic landmarks and adjunctive fluoroscopy can be challenging. However, we noticed that screw placement did not have known adverse outcomes in this cohort. There was a low rate of spinal canal penetration and no aortic impingement.

## Data Availability

Data would be available upon request.
